# Impact of Diabetes Mellitus on Outcomes of Patients With Knee Osteoarthritis Who Underwent Knee Arthroplasty: An Analysis of the Nationwide Inpatient Sample

**DOI:** 10.7759/cureus.8902

**Published:** 2020-06-29

**Authors:** Pius E Ojemolon, Hafeez Shaka, Ehizogie Edigin, Trisha Marie A Gomez, Precious Eseaton, Jeremiah Bello, Clark Azubuike, Omokunmi P Adekola

**Affiliations:** 1 Anatomical Sciences, St. George's University, St. George's, GRD; 2 Internal Medicine, John H. Stroger, Jr. Hospital of Cook County, Chicago, USA; 3 Internal Medicine, University of Benin, Benin City, NGA; 4 Medicine, University of Benin, Benin City, NGA; 5 Emergency Medicine, University of Benin, Benin City, NGA; 6 Medicine and Surgery, University of Ibadan, Ibadan, NGA

**Keywords:** knee osteoarthritis, knee replacement, diabetes mellitus, perioperative outcomes

## Abstract

Background

Knee arthroplasty is one of the most common reasons for hospitalizations in the United States. Diabetes mellitus is thought to be associated with adverse perioperative outcomes. We sought to demonstrate the effect of comorbid diabetes on hospitalizations involving patients with knee osteoarthritis who had knee arthroplasty.

Materials and methods

Data was obtained from the Nationwide Inpatient Sample (NIS) for 2016 and 2017. ICD-10 codes were used to obtain a cohort of patient who were principally admitted for knee osteoarthritis who underwent knee arthroplasty. The patients were further divided according to diabetic status. The primary outcome compared inpatient mortality. Secondary outcomes included mean length of hospital stay, total hospital charges, presence of secondary diagnoses on discharge of acute kidney injury, surgical site infection, sepsis, thromboembolic events, non-ST segment elevation myocardial infarction (NSTEMI).

Results

Patients with diabetes mellitus had a lower adjusted odds ratio for mortality (aOR: 0.45 95% CI: 0.221 - 0.920, p = 0.029), with no significant difference in total hospital charges and length of hospital stay. Interestingly, patients with diabetes had lower odds of NSTEMI; 0.53 (95% CI: 0.369 - 0.750, p < 0.001) sepsis; 0.64 (95% CI: 0.449 - 0.924, p = 0.017) and DVT; 0.67 (95% CI: 0.546 - 0.822, p < 0.001).

Conclusion

Uncomplicated diabetes mellitus is not associated with adverse outcomes in patients hospitalized with knee osteoarthritis who had knee arthroplasty.

## Introduction

Osteoarthritis (OA) is a chronic, degenerative disease of the joints that primarily affects the cartilage [1]. OA is the most common degenerative joint disease globally and represents a substantial health burden with notable implications for the individuals and healthcare systems affected. OA is a major cause of pain and disability amongst adults worldwide, with global estimates suggesting that 250 million people are currently affected [2]. The knee joint is most commonly affected by OA, and individuals suffering from knee OA commonly experience pain, stiffness and associated loss of function in the affected knees [2, 3]. Knee OA is the most common reason for total knee arthroplasty (KA) in the United States [4].

Diabetes mellitus (DM) refers to a group of metabolic diseases characterized by aberrant glucose metabolism and persistent hyperglycemia resulting from defects in insulin secretion, insulin action, or both [5].

OA and DM are rising in prevalence globally and presently affects millions. They both share a major risk factor, obesity, but DM is beginning to be recognized as a probable independent risk factor for OA [6-8]. Peri-operative hyperglycemia has been established as a poor prognostic factor following surgery [9-11]. However, there is a paucity of studies comparing outcomes of diabetic and non-diabetic patients with knee OA who underwent KA.

We aimed to investigate possible disparities in mortality and post-surgical complications between these two patient populations.

## Materials and methods

Design and data source

This study design was a retrospective cohort study involving adult hospitalizations principally for knee OA who had knee replacement procedure done in the US between January 1, 2016 and December 31, 2017. Data was sourced from the Nationwide Inpatient Sample (NIS) database for 2016 and 2017. The NIS is a database of hospital inpatient stays derived from billing data submitted by hospitals to statewide data organizations across the US, covering more than 97% of the U.S. population [12]. It approximates a 20% stratified sample of discharges from U.S. community hospitals, excluding rehabilitation and long-term acute care hospitals. This dataset is weighted to obtain national estimates [13]. Both the 2016 and 2017 database are entirely coded using the International Classification of Diseases, Tenth Revision, Clinical Modification/Procedure Coding System (ICD-10-CM/PCS). In the NIS, diagnoses are divided into two separate categories: principal diagnosis and secondary diagnoses. A principal diagnosis was the main ICD-10 code for the hospitalization. Secondary diagnoses were any ICD-10 code other than the principal diagnosis.

Study population

We queried the NIS 2016 and 2017 database for patients 18 years and above who had a principal discharge diagnosis of knee OA and underwent any knee replacement procedure. Patients were excluded if they were younger than 18 years of age, or if knee OA was listed as a secondary diagnosis. This cohort was further divided based on the presence of at least one secondary discharge diagnosis of diabetes mellitus types 1 and 2. We used ICD-10-CM/PCS codes to obtain the cohort and these can be found in the appendix.

Outcome measures

The primary outcome was inpatient mortality among patients principally admitted for knee OA who had any knee replacement procedure. Secondary outcomes in this population include discharge diagnoses of non-ST segment elevation myocardial infarction (NSTEMI), sepsis, post procedure site infection, pneumonia, acute kidney failure (AKI), deep vein thrombosis (DVT), pulmonary embolism (PE), need for transfusion of blood products, complications involving orthopedic devices as well as mean length of hospitalization and mean total hospital charges.

Statistical analysis

We analyzed the data using Stata® Version 16 software (StataCorp, Texas, USA). All analyses were conducted using the weighting samples for national estimates in adjunct with Healthcare Cost and Utilization Project regulations for using the NIS database. Comorbidities were calculated as proportions of the cohort and Chi squared test was used to compare these characteristics between the diabetic and non-diabetic subgroups. Chi squared test was also used to compare tabulated grouped variables, e.g. race, insurance type and hospital bed size. Multivariate regression analysis was done to adjust for possible confounders while calculating the primary and secondary outcomes. These were found from literature review. A univariate screen was done to further confirm these factors. All p-values were 2 sided, with 0.05 as the threshold for statistical significance.

Ethical considerations

The NIS database has been completely stripped of possible patient identifiers. Since 2012, the NIS has also removed state level and hospital identifiers. This has enhanced patient protection and anonymity. This study was exempt from Institutional Review Board approval.

## Results

Patient characteristics

The combined NIS database for 2016 and 2017 contained over 71 million hospital discharges of which 1,479,010 (2.1%) were included in the study. These patients were adults with a principal discharge diagnosis of knee OA who underwent KA. Of this group, 317,975 (21.5%) had DM, defined by ICD-10 codes.

Patients with DM were significantly older (mean age of 67.1 vs 66.4 years, p < 0.001) and had more co-morbidities including hypertension (72.5 vs 56.7%, p < 0.001), smoking history (30.5 vs 28.9%, p < 0.001), congestive heart failure (4.7 vs 2.0%, p < 0.001) and chronic kidney disease (9.1 vs 3.5%, p < 0.001) compared with patients without DM. Table 1 demonstrates the demographic characteristics of the study.

**Table 1:** Patient and hospital characteristics of diabetic vs non-diabetic patients with knee osteoarthritis who had knee arthroplasty CHF: Congestive heart failure, CKD: Chronic kidney disease, CVA: Cerebrovascular accident, IHD: Ischemic heart disease

Variable	Overall n = 1,479,010	Diabetic n = 317,975 (21.5)	Non-diabetic n = 1,161,035 (78.5)	p
Patient characteristics				
Age, mean years	66.5	67.1	66.4	
Women	(61.5)	(58.6)	(62.3)	<0.001
Racial distribution				<0.001
White	(81.8)	(74.6)	(83.7)	
Black	(8.0)	(11.3)	(7.1)	
Hispanic	(6.1)	(8.6)	(5.4)	
Others	(4.1)	(5.5)	(3.8)	
Insurance type				<0.001
Medicaid	(58.8)	(63.7)	(57.4)	
Medicare	(4.4)	(4.9)	(4.3)	
Private	(36.3)	(31.0)	(37.8)	
Uninsured	(0.5)	(0.4)	(0.5)	
Charlson Comorbidity Index score				<0.001
0	(56.8)	(0.0)	(72.3)	
1	(26.5)	(54.8)	(18.7)	
2	(10.0)	(24.0)	(6.2)	
≥3	(6.7)	(21.2)	(2.8)	
Median annual income in patient’s zip code, US$				<0.001
1-43,999	(22.1)	(26.0)	(21.0)	
44,000-55,999	(26.6)	(28.0)	(26.2)	
56,000-73,999	(27.1)	(26.2)	(27.4)	
≥74,000	(24.2)	(19.8)	(25.4)	
Co-morbidities				
Hypertension	(60.1)	(72.5)	(56.7)	<0.001
Smoking history	(28.9)	(30.5)	(28.5)	<0.001
CHF	(2.6)	(4.7)	(2.0)	<0.001
CKD	(4.7)	(9.1)	(3.5)	<0.001
Dyslipidemia	(45.4)	(63.2)	(40.5)	<0.001
Obesity	(29.5)	(40.5)	(26.5)	<0.001
Chronic IHD	(11.2)	(18.1)	(9.4)	<0.001
Prior CVA	(0.4)	(0.6)	(0.3)	<0.001
Atherosclerosis	(0.5)	(0.7)	(0.5)	<0.001
Liver disease	(1.2)	(1.9)	(1.0)	<0.001
Hospital characteristics				
Hospital region				<0.001
Northeast	(17.7)	(16.3)	(18.1)	
Midwest	(26.7)	(26.9)	(26.6)	
South	(35.8)	(38.9)	(35.0)	
West	(19.8)	(17.9)	(20.3)	
Hospital bed size, no. (%)				<0.001
Small	(30.2)	(29.0)	(30.5)	
Medium	(28.7)	(29.2)	(28.6)	
Large	(41.1)	(41.8)	(40.9)	
Urban location	(89.6)	(89.0)	(89.8)	<0.001
Teaching hospital	(60.3)	(59.8)	(60.4)	0.038

Primary outcome: in-hospital mortality

The in-hospital mortality for patients with knee OA who had KA was 0.024%. There were 355 reported deaths. Patients with DM did not have higher odds of in-hospital mortality (adjusted odds ratio [aOR]: 0.45, 95% confidence interval [CI]: 0.221-0.920, p = 0.029) when adjusted for co-morbidities using multivariate logistic regression analysis.

Secondary outcomes

The total length of hospital stay, and the total hospital charges between the DM and non-DM group were compared using multivariate linear regression model. There was no mean difference in length of hospital stay (0.005, CI: [-0.017] - 0.028, p = 0.638) in days and hospital charge (276 (-220 - 771) in USD. DM patients had lower aOR of having NSTEMI (0.53, 95% CI: 0.369 - 0.750, p < 0.001), sepsis (0.64, 95% CI: 0.449 - 0.924, p = 0.017), deep vein thrombosis (0.67, 95% CI: 0.546 - 0.822, p < 0.001), and need for transfusion of blood products (0.90, 95% CI: 0.823 - 0.993, p = 0.034. Detailed outcomes are provided in Table 2.

**Table 2:** Clinical outcomes in hospitalizations comparing diabetic vs non-diabetic patients principally admitted for knee osteoarthritis who had knee arthroplasty * = Statistically significant, # = mean difference aOR: adjusted odds ratio, CI: Confidence interval, NSTEMI: Non-ST segment elevation myocardial infarction.

Outcome	Diabetic	Non-diabetic	aOR (95% CI)	p
Primary outcome				
In hospital mortality	105	250	0.45 (0.221 - 0.920)	0.029*
Secondary outcomes				
Length of stay, mean days	2.6	2.3	0.005^#^ (-0.017 - 0.028)	0.638
Total hospital charges, mean USD	61444	58817	276^#^ (-220 - 771)	0.275
NSTEMI	320	505	0.53 (0.369 - 0.750)	<0.001*
Sepsis	340	780	0.64 (0.449 - 0.924)	0.017*
Pneumonia	1105	1955	1.00 (0.815 - 1.224)	0.992
Post procedure infection	160	305	1.63 (0.9260 - 2.86)	0.091
Deep vein thrombosis	965	3200	0.67 (0.546 - 0.822)	<0.001*
Pulmonary embolism	830	2390	0.99 (0.779 - 1.262)	0.941
Complications from orthopedic device	695	3075	0.97 (0.764 - 1.243	0.832
Need for transfusion of blood products	6635	17575	0.90 (0.823 - 0.993)	0.034*
Acute kidney failure	10455	14285	1.03 (0.952 - 1.117)	0.445
Pressure related injury	108	255	1.20 (0.713 - 2.018)	0.494

## Discussion

The prevalence of DM in our study population was 21.5%, which is more than twice the estimated prevalence of DM in the general US population (10.5%) [14]. This is similar with the findings of other studies, pointing towards increased risk of OA in DM patients [6,7]. Over 60% of the patients with knee OA were females. This is in common with other recent studies which have associated the female gender with an increased risk of knee osteoarthritis [15-17].

Our study showed that uncomplicated DM does not have significant impact on outcomes during hospitalizations of patients with knee OA who had knee replacement. Diabetes is associated with increased risk and accelerates the progression of OA in several studies, although the confounding effect of concomitant obesity was not considered [6-8]. Schett et al. found DM to be an independent risk factor of severe OA with a hazard ratio (HR) of 2.1 (P = 0.023) after adjusting for body mass index (BMI) [18].

Findings from studies conducted by Jämsen et al. and Memtsoudis et al. indicate that increased in-hospital mortality after hip and knee replacement has been linked to renal disease, cerebrovascular disease, and DM [19,20]. This is at variance with our study, likely as a result of known association of DM with other chronic medical disease, especially in cases of complicated DM.

There was a statistically significant lower odds of having NSTEMI, sepsis, DVT and the need for transfusion post KA as secondary discharge diagnoses in patients with DM who had KA. There were no significant differences in odds of pneumonia, post-procedure infection, PE, AKI, pressure-related injury or complications from orthopedic devices between both cohorts. Comparatively, a study conducted by Maradit Kremers et al. showed a higher risk of prosthetic joint infection within one year of elective primary hip and knee arthroplasty in patients with either a diagnosis of DM or perioperative hyperglycemia (HR 1.55 with 95% CI of 1.11-2.16). However, just like this study, these effects did not remain statistically significant when adjusted for comorbidities for BMI, American Society of Anesthesiologist (ASA) score, and operative time [21]. Similar findings were obtained from a study conducted by Schwartz et al. which showed that although hemoglobin A1C has often been used as a marker of diabetes control for risk stratification prior to joint arthroplasty, it has not been strongly linked to an elevated risk of postoperative complications [22].

This study also showed that there was no significant difference in the major indices available to measure health care utilization which are total hospital charges and length of hospital stay.

This study has several strengths. First, it utilized information from the NIS, a large nationwide dataset to provide a large sample size to compare mortality outcomes even in the setting of extremely low inpatient mortality rate associated elective joint arthroplasty. Second, the nature of the database provides insight on the comparison of baseline demographics and hospital outcomes between knee OA hospitalizations with or without DM to statistically significant levels.

There are some limitations to this study. There was non-randomization as a result of the nature of the NIS database. This is highlighted by the difference in distribution of comorbidities in both groups. There is a possibility of interpersonal variation with coding, as the NIS is an administrative database that uses ICD-10 codes to characterize diagnoses and hospitalization events. Thus, we cannot determine if underlying disease severity of OA affected the outcome of the patients. Another limitation is that laboratory and radiologic data like blood glucose readings, or knee X-rays which could indicate underlying disease severity and inflammatory activity are not available in the NIS database.

## Conclusions

Among patients with knee OA who had KA, uncomplicated DM does not negatively impact in-hospital outcomes. The lower odds of complications among DM patients raises the possibility of other health benefits as a result of DM-directed management. Further research is needed in identifying these protective factors.

## Appendices

**Table 3:** ICD-10 CM/PCS codes

Variable	Diagnostic code
Knee osteoarthritis	M17
Knee replacement procedures	0SRC, 0SRD, 0SRT, 0SRU, 0SRV, 0SRW
Diabetes mellitus	E10, E11
Post procedural infection	T814
Pressure ulceration	L89
Sepsis	A40, A41, R652, T8112
Bacterial pneumonia	J13, J14, J15, J16
Non-ST elevation myocardial infarction	I214
Venous thromboembolism	I82
Pulmonary embolism	I26
Complications of internal orthopedic prosthetic devices	T84
Transfusion of blood products	302
Acute kidney failure	N17
